# Nrf2 Mediates Metabolic Reprogramming in Non-Small Cell Lung Cancer

**DOI:** 10.3389/fonc.2020.578315

**Published:** 2020-11-26

**Authors:** Jiangang Zhao, Xu Lin, Di Meng, Liping Zeng, Runzhou Zhuang, Sha Huang, Wang Lv, Jian Hu

**Affiliations:** Department of Thoracic Surgery, The First Affiliated Hospital, School of Medicine, Zhejiang University, Hangzhou, China

**Keywords:** nuclear factor erythroid-2–related factor-2, metabolic reprogramming, non-small cell lung cancer, Kelch-like ECH-associated protein 1, reduction-oxidation balance

## Abstract

Nuclear factor erythroid-2–related factor-2 (NFE2L2/Nrf2) is a transcription factor that regulates the expression of antioxidant genes. Both Kelch-like ECH-associated protein 1 (Keap1) mutations and Nrf2 mutations contribute to the activation of Nrf2 in non-small cell lung cancer (NSCLC). Nrf2 activity is associated with poor prognosis in NSCLC. Metabolic reprogramming represents a cancer hallmark. Increasing studies reveal that Nrf2 activation promotes metabolic reprogramming in cancer. In this review, we discuss the underlying mechanisms of Nrf2-mediated metabolic reprogramming and elucidate its role in NSCLC. Inhibition of Nrf2 can alter metabolic processes, thus suppress tumor growth, prevent metastasis, and increase sensitivity to chemotherapy in NSCLC. In conclusion, Nrf2 may serve as a therapeutic target for the treatment of NSCLC.

## Introduction

Lung cancer is the leading cause of cancer-related death worldwide (1). Non-small cell lung cancer (NSCLC) is the most common lung cancer type, including adenocarcinoma, squamous carcinoma, adenosquamous carcinoma, and large cell lung cancer, etc. During the past two decades, targeted therapy and immunotherapy have resulted in dramatic tumor response compared to conventional chemotherapy and radiotherapy in patients with NSCLC. Nevertheless, numerous patients can benefit from neither conventional and nor novel therapeutic approaches. More treatments are in need to offer possible solutions to patients with dead ends.

Metabolic reprogramming is a hallmark of cancer (2). Half a century ago, Otto Warburg firstly observed that unlike normal cells, tumor cells preferentially utilize glycolysis in an oxygen-rich environment, as called Warburg Effect. Recent studies revealed that cancer cells use multiple metabolic pathways to drive and maintain their malignant phenotypes. This metabolic shifting in cancer cells is regarded as metabolic reprogramming. For example, tumor cells consume glucose, lactate, pyruvate, hydroxybutyrate, acetate, glutamine, and fatty acids at much higher rates than their non-tumor equivalents, which enables tumor cells to produce enough ATP as an energy source and offer intermediate products for biosynthesis. Meanwhile, these processes also generate toxic substances. Therefore, maintenance of the reduction-oxidation (redox) balance is indispensable to promote tumor cell growth and metastasis (3, 4). Metabolic reprogramming is highly active and shows heterogeneity among patients with NSCLC (5). Thus, targeting the altered metabolic pathways holds promise as a novel strategy in NSCLC.

The Kelch-like ECH-associated protein 1-nuclear factor erythroid-2–related factor 2 (Keap1-Nrf2) signaling pathway plays a vital role in the protection against oxidative stress, such as ROS. Under normal conditions, Nrf2 is constitutively ubiquitinated by Keap1 and Cullin-3 E3 ligase and degraded by the 26S proteasomal pathway (6). Under oxidative stress conditions, Keap1-mediated Nrf2 degradation is abrogated by the oxidation of Keap1. Keap1 has more than 20 free sulfydryl (-SH) groups in its constituent cysteine residues, which are highly reactive functional groups acting as stress sensors. Intracellular ROS can modify Keap1 cysteine residues. These modifications result in a conformational change of Keap1, thereby preventing the proteasomal degradation of Nrf2 (7). Increased Nrf2 translocates to the nucleus, heterodimerizes with small Maf proteins, and induces the transcriptional activation of target genes with antioxidant response elements (AREs) in their gene regulatory regions (8). Thus, the Keap1-Nrf2 pathway maintains intracellular redox homeostasis.

Loss-of-function mutations of Keap1 and gain-of-function mutations of Nrf2 have been observed in lung cancer. Frank R et al. used next-generation sequencing (NGS) to analyze the tumor tissue of 1,391 patients with non-small cell lung carcinoma (NSCLC) and found that Keap1 mutations occurred in 11.3% (n = 157) and NRF2 mutations occurred in 3.5% (n = 49) of NSCLC patients (9). Both loss-of-function mutations of Keap1 and gain-of-function mutations of Nrf2 result in constitutive Nrf2 activity. Besides, other mechanisms, such as epigenetic hypermethylation of the promoter of Keap1, may also contribute to the accumulation and activation of Nrf2 in lung cancer. Chien MH et al. analyzed 238 lung cancer specimens using immunohistochemistry and revealed an inverse correlation between Nrf2 and Keap1 expression. They reported that patients with lung adenocarcinoma exhibiting a high expression level of Keap1 and a low expression level of Nrf2 had significantly better overall survival and disease-free survival than patients exhibiting a low expression level of Keap1 and a high expression level of Nrf2 (10).

Although Nrf2 has been widely recognized as an oxidative stress regulator, increasing studies have shown its role in manipulating cancer metabolism (2, 11–13). Nrf2 regulates multiple key metabolic genes in cancer cells directly through its ARE function or indirectly through the crosstalk with other transcription factors. Notably, Nrf2 controls multiple key enzymes in the network of metabolism, thereby altering the metabolic cascade, including carbohydrates, amino acids, lipid, and nucleic acid metabolism. As a result, Nrf2 alters the redox balance, providing a new plateau that favors tumor progression in NSCLC.

In this review, we outline the role of Nrf2 in cancer metabolism regulation, elucidate the crosstalk between Nrf2 and metabolic reprogramming, and discuss the potential therapeutic targets in NSCLC.

## Nrf2-Mediated Metabolic Reprogramming in Lung Cancer

As mentioned above, Nrf2 accumulates in the nucleus, heterodimerizes with small Maf proteins, and induces the expression of genes harboring an ARE in their regulatory regions. Well-known genes regulated by Nrf2 include NAD(P)H dehydrogenase, quinone 1 (NQO1), glutamate-cysteine ligase, modifier subunit (GCLM), heme oxygenase (decycling) 1 (HMOX1), etc. (14–16).

Namani A et al. analyzed Chip-Seq and microarray data of A549 cells and performed KEGG pathways analysis. They found that target genes of Nrf2 are involved in metabolic pathways, including porphyrin and chlorophyll metabolism, glycolysis/gluconeogenesis, pentose phosphate pathway (PPP), pyruvate metabolism, fructose, and mannose metabolism, metabolism of xenobiotics by cytochrome P450, glutathione metabolism, arachidonic acid metabolism, ascorbate, and aldarate metabolism, pentose and glucuronate interconversions, and steroid hormone biosynthesis (17).

### Reactive Oxygen Species

Reactive oxygen species (ROS) are produced in eukaryotic cells through aerobic metabolism, including superoxide, hydrogen peroxide, hydroxyl radical, singlet oxygen, peroxyl radical, alkoxyl radical, lipid hydroperoxide, peroxynitrite, and hypochlorous acid (18). They can be generated both in the cytoplasm by NADPH oxidases (NOX), xanthine oxidase, cyclooxygenases, and cytochrome P450 enzymes, and in the mitochondria by the respiratory chain, monoamine oxidases (MAOs), p66shc, and NOX4 (19). ROS are important signaling molecules. The tight regulation of ROS levels is crucial for cell fate determination. Under normal conditions, moderate ROS enhances cell proliferation and differentiation. However, when ROS production increases rapidly or ROS elimination decreases heavily, cells encounter a condition known as oxidative stress. Excessive ROS cause oxidative damage to proteins, lipids, and DNA (20). In NSCLC, abundant energy production and synthesis of biological molecules are indispensable to maintain the malignant behavior of tumor cells. The levels of ROS are thought to be well-regulated in order to drive tumor progression in NSCLC.

Nrf2 is often considered as the main regulator of ROS (21). Increased levels of ROS in the cytoplasm lead to disassociation of Keap1 and Nrf2. Thereby, Nrf2 enters the nucleus to function as an anti-oxidative transcription factor (22). Nrf2-regulated genes prevent the oxidation of Cys residues directly or indirectly through the cascade reaction of enzymes to reduce the ROS modification of Cys. The knockdown of Nrf2 in NSCLC cell lines can increase the endogenous level of ROS dramatically and enhance their sensitivity to radiation therapy (23). Nrf2-mediated ROS homeostasis results in paclitaxel chemoresistance via PI3K/Akt pathway in NSCLC (24). Besides, through the regulation of ROS, Nrf2 also affects cholesterol synthesis by preventing the silencing of 3-hydroxy-3-methylglutaryl-CoA (HMG-CoA) reductase, the rate-limiting enzyme of cholesterol synthesis, by the inhibition of ROS (25).

### NADPH Synthesis

Nicotinamide adenine nucleotide phosphate (NADPH), one of the most important elements of cell metabolism, is required in lipid, glucose, and nucleotide biosynthesis and regulation (26, 27). Nrf2 plays a crucial role in NADPH metabolism. Most NADPH-related genes are regulated by Nrf2, including four NADPH generating enzymes glucose-6-phosphate dehydrogenase (G6PD), isocitrate dehydrogenase (IDH1), malic enzyme 1 (ME1), and phosphogluconate dehydrogenase (PGD). The knockdown of Nrf2 expression decreases the NADPH level and the NADPH/NADP^+^ ratio (27, 28). Further studies suggested that Nrf2 related chemoresistance can be induced when the degradation of NOX2 is interfered, thereby less ROS is produced to perform the cytotoxic effect and become chemoresistance (29). In NSCLC, NADPH-related genes, NOX2, and NOX4 are overexpressed, which play as a metabolic promotor in glycolysis, glutamine, and lipid metabolism, thereby resulting in tumor progression and resistance (30, 31).

### Pentose Phosphate Pathway

The PPP is crucial for cancer, not only providing NAPDH for biosynthesis but also working as the major nucleic acid supplier (32). As mentioned, Nrf2 regulates the major NADPH produce enzyme, G6PD, and PGD, which are from the oxidative phase of PPP (11). This suggests the ability of Nrf2 to control the NADPH level in cell metabolism. The non-oxidative stage of PPP is also regulated by Nrf2, where transaldolase 1 (TALDO1) is directly regulated by Nrf2 with its ARE function (33, 34). In Kras^G12D^ mutated lung cancer, Keap1 mutation leads to high Nrf2 levels thereby positively regulating TALDO1 levels to promote oncogenesis and cancer development via PPP levels. Moreover, the adoption of PPP inhibitor 6-aminonicotinamide (6-AN) can reverse cancer-promoting events and prevent cancer progression in vivo (35).

### Glutathione Regulation

As a powerful antioxidant, glutathione (GSH) plays a vital role in tumor growth and metastasis. Tumor samples of NSCLC have higher levels of GSH compared with normal lung tissues, along with higher GSH uptake ability (36). Moreover, patients with NSCLC who underwent standard chemotherapy were found with higher levels of GSH in plasma (37). In the scenario of radiotherapy, GSH acts as the key protector of radiation injury. Nrf2 is one of the key regulators of GSH metabolism. GSH synthesis enzyme glutamate-cysteine ligase and glutathione synthetase are target genes of Nrf2 (38, 39). Glutaminase (GLS), which can catalyze glutamine into glutamate is also promoted by Nrf2 (11). Besides, the expression of SLC7A11, the light chain subunit of the Xc- antiporter system (xCT) is activated by Nrf2. xCT performs as the translocator of cystine and glutamate, while cystine is imported into the cell by xCT, and glutamate is exported (40). In Keap1 mutant NSCLC, high levels of Nrf2 enhance the cell dependency on GSH. A high level of Nrf2 expression can result in stronger radiotherapy resistance through the effect of GSH (23).

### Lipid Metabolism

Although the main physical lipid metabolism organ is the liver, it plays a dramatic role in the functioning of the lung, especially on the surface of pulmonary alveoli. Lipid metabolism is also involved in NSCLC development. Studies showed that lipid synthesis is the metabolic liability of lipid metabolism in NSCLC (41). Different from normal tissue, NSCLC shows an enhanced lipid synthesis ability to favor its lipid demand due to rapid cell proliferation. When cancer cells are in a glucose deprived scenario, lipid oxidation becomes the main source of ATP, NADPH, and FADH2 (42).

In general, Nrf2 plays as a negative regulator to lipid levels. Nrf2 regulates several lipases at the lung site, including lipase, member H (LIPH), phospholipase A2, group vii (PLA2G7), and patatin-like phospholipase domain containing 2 (PNPLA2) (43). Biologically these lipases are involved in the starvation response to triglycerides and phospholipids degradation. On the other hand, stearoyl CoA desaturate 1 (SCD1), the rate-limiting enzyme in lipid synthesis, is reported negatively regulated by Nrf2, no matter in genetic and pharmacological activated model (44). The knockout of Nrf2 shows high levels of SCD1 expression, thereby a higher triglyceride level is observed (45). SCD1 can deactivate AMP kinase (AMPK), thus activate acetyl-CoA carboxylase 1 (ACC1) activity, resulting in the formation of complex lipids (46). ACC1 is another key enzyme in de novo lipid synthesis, which is also regulated by Nrf2 (27). ACC1 catalyzes acetyl-CoA to malonyl-CoA. In the scenario of NSCLC, the ACC family is highly expressed in many cell lines. ACC is observed as participated in tumor proliferation (41). Inhibition of ACC1 expression by CRISPR-Cas9 system shows markedly proliferation defects, but this phenomenon can be rescued by the treatment of outsourcing fatty acid, suggesting the irreplaceable role of ACC1 in NSCLC development (47). Moreover, increasing of ACC1 will lead to apoptosis of cisplatin-resistant lung cancer cells (48).

In addition, Nrf2-mediated downregulation of ACC1 and SCD1 will decrease the levels of malonyl-CoA, thereby result in higher fatty acid oxidation. Malonyl-CoA is a natural inhibitor of carnitine palmitoyltransferase 1 (CPT1), which mediates lipid transportation into mitochondria and undergo oxidation (49). CPT1 is considered as the rate-limiting enzyme of mitochondrial oxidation of lipids. Another Nrf2-mediated translocase CD36 also participated in lipid oxidation in the same manner (50). Other Nrf2 target genes that regulate lipid oxidative include nuclear receptor retinoid X receptor alpha (RXRa) and peroxisome proliferator-activated receptor-gamma (PPARg) (51). In summary, Nrf2 can mediate lipid oxidation through its downstream gene expression directly or indirectly manipulate mitochondrial transportation of lipids.

### Glycolysis and Gluconeogenesis

The Warburg effect depicts the dependency of the glycolysis of tumor cells. In NSCLC, inhibition of Nrf2 leads to a low glycolysis condition in cell metabolism, suggesting the role of Nrf2 in maintaining basic energy supply in NSCLC. As mentioned in *Pentose Phosphate Pathway*, whether glucose will be utilized through glycolysis or PPP will be affected by Nrf2 since its downstream control of G6PD activity, the PPP determinator. Another target regulated by Nrf2 in glycolysis is pyruvate kinase (PK), the key enzyme of glycolysis which catalyzes phosphoenolpyruvate to pyruvate (44). However, affecting PK activity, the rate-limiting enzyme of glycolysis will channel glucose metabolism toward PPP, which will lead to phospholipid synthesis. In the scenario of cancer, PK inhibition leads to PPP promoting will end up with carcinogenesis related biosynthesis and redox defense through high NADPH level (52).

Phosphoenolpyruvate carboxykinase (PCK1) is the key enzyme of gluconeogenesis, which can regulate Nrf2 level negatively in cancer cell lines (52). Nrf2 will thereby regulate thioredoxin reductase 1 (TXNRD1) to induce cell proliferation. The downstream targets of Nrf2 function also include several gluconeogenesis genes. Studies about in vivo diabetic model suggested that Nrf2 is associated with the cAMP-CREAB pathway, which negatively regulates gluconeogenesis through FBP1 (fructose-1,6-bisphosphatase 1), PGC1a (peroxisome proliferator-activated receptor g coactivator 1-a), and NR4A2 (nuclear receptor subfamily 4, group A, member 2) (53).

### Nrf2 in Iron Metabolism and Ferroptosis

Iron is one of the microelements of the human body, which is well known as a functioning unit in heme as an oxygen carrier. Iron also participates in several metabolic diseases. Lack of iron will cause anemia, hypotrichosis, while overload of iron will result in hepatic fibrosis or hepatic cancer (54). Nrf2 was firstly identified as an erythroid gene regulator, where ATP binding cassette subfamily B member 6 (ABCG6) and Ferrochelatase (FECH) are enzymes that participated in heme synthesis. Biliverdin reductase A and B (BLVRA and BLVRB), HMOX1 are downstream genes of Nrf2 in heme catabolism. ferritin heavy chain (FTH1) and ferritin light chain (FTL) is in charge of iron pool homeostasis (55). Lignitto L et al. showed that heme causes the degradation of Bach1, a pro-metastatic transcription factor by promoting its interaction with the ubiquitin ligase Fbxo22. They found that Nrf2 overexpression in lung cancer promotes the stabilization of Bach1 by inducing HO1, the enzyme catabolizing heme (16). Wiel C et al. found that long-term supplementation with the antioxidants N-acetylcysteine and vitamin E promotes lung cancer metastasis by reducing levels of free heme and stabilizing BACH1. Moreover, they revealed that BACH1 activates the transcription of Hexokinase 2 and GAPDH and increases glucose uptake ability, glycolysis rates, and lactate secretion, resulting in glycolysis-dependent metastasis of lung cancer cells (56). These studies revealed that Nrf2 participates in metabolic reprogramming in NSCLC.

In the past decade, increasing studies suggest the appealing role of iron homeostasis in cancer development and progression (45, 57–59), while Nrf2 plays a critical role in shifting iron metabolism toward the cancer phase (55). Studies have shown HMOX1 has a non-enzyme function to promote chemoresistance in lung cancer (60). Nrf2 and HMOX1 synergy will induce angiogenesis through the activation of thymidine phosphorylase in lung cancer cells (61). FTH1 and FTL lead iron deficiency will start cascade events to proliferate gene activation and tumor necrosis factor α (TNFα) tolerance (55).

Recently, an iron-dependent, programmed cell death was discovered and named ferroptosis (62). The accumulation of free iron in cell plasma will cause lipid peroxidation through Fenton Reaction, thus lipid peroxidation will sabotage the integrity and functions of the membrane system and lead to cell death (63). The morphology of ferroptosis will start with mitochondrial shrinkage. Lipid peroxidation in ferroptosis can also be initiated by ROS, especially hydroxyl radical and hydroperoxyl radical. By contrast, lipid peroxidation can be reduced by glutathione peroxidases, such as glutathione peroxidase 4 (GPX4). Ferroptosis has been confirmed as one of the major pathways related to chemoresistance and radiotherapy resistance in lung cancer (64). During lung cancer progression, ferroptosis also has a role as a selector to tolerate a high oxygen environment (65).

In general, Nrf2 plays as a protector of lung cancer cells from ferroptosis. Nrf2 regulated iron pool-related genes including FTH1 and FTL can control ferroptosis through the level of free iron in the cytosol. On the other hand, the NADPH level regulated by Nrf2 will also affect the process of ferroptosis through the GSH level. Solute carrier family 7 member 11/System xCT (SLC7A11) promoted by Nrf2 can increase intracellular cysteine, which also leads to a high GSH level to protect lipid peroxidation. A recent study shows antioxidator NOX4, downstream gene of Nrf2, can reverse lipid peroxidation with NADPH.

### Mitochondrial Metabolism

The role of Nrf2 in mitochondrial structure and function has attracted extensive interests recently. Knockdown of Nrf2 results in the decrease of ATP production in cancer cells (66). By contrast, activation of Nrf2 leads to increased ATP production, high basal mitochondrial membrane potential, and low GSH level in lung cancer cell lines (65, 66). Nrf2 modulates mitochondrial metabolism not only by manipulating substrates providing, such as NADPH and FADH2, but also directly by regulating genes that participate in the respiratory chain complex (67). Nrf2 positively regulates the transcription of nuclear respiratory factor 1, which is the key regulator of the expression of respiratory complexes (68, 69), including subunits of complex IV cytochrome c NADH Dehydrogenase 1 Alpha Subcomplex (NDUFA4), cyclooxygenase-2 (COX2) and cyclooxygenase 4I1 (COX4l1) (70) Moreover, studies suggest that Nrf2 also participate in mitochondria biogenesis (69, 71). Under oxidative stress, Nrf2 controls the expression of the gene encoding uncoupling protein 3 (UCP3), which maintains an inner mitochondrial membrane function to relieve superoxide production (72). These studies indicate that the intricate role of Nrf2 in regulating the bio function of mitochondria. However, whether Nrf2-mediated mitochondrial metabolism reprogramming affects NSCLC growth and progression remains unclear.

### Therapeutic Approaches

As we discussed above, Nrf2 has been recognized as an important driver in the development and progression of NSCLC. Therefore, targeting Nrf2 and its downstream molecules is a hot research topic now and various types of Nrf2 inhibitors have been developed in the field of cancer therapy. Singh A et al. developed a small molecule inhibitor of Nrf2, ML385 (73). ML385 can bind to Neh1, the Cap “N” Collar Basic Leucine Zipper (CNC-bZIP) domain of Nrf2, and interfere the binding with the V-Maf Avian Musculoaponeurotic Fibrosarcoma Oncogene Homologue G (MAFG) to regulatory bind with target DNA sequences. In vitro and in vivo experiments, ML385 selectively inhibits the proliferation of NSCLC cells with Keap1 mutation. Further study showed that ML385 substantially enhances paclitaxel, doxorubicin, or carboplatin cytotoxicity against lung cancer cells with Keap1 mutation (73). Romero R et al. found that KRAS-driven lung tumors that bear Keap1 or Nrf2 mutations depend heavily on glutaminolysis for carcinogenesis (74). Pooled CRISPER/Cas9 sgRNA library revealed that Solute carrier family 1 member 15 (SLC1A5), a glutamine transport enzyme, is the key element responsible for this effect. CB-839, a glutaminase inhibitor, inhibits the growth of KARS and Keap1 co-mutated lung cancer (75, 76). Another small molecule with anti-cancer efficacy is ND-646, an ACC inhibitor (77). ND-646 can bind to the BC domain that contains phosphorylated AMPK interaction target, thereby preventing ACC from dimerization and downstream activation to fatty acid synthesis. In vivo studies showed that ND-646 inhibits the growth of NSCLC cells. Moreover, the detailed analysis demonstrated that ND-646 has better anti-cancer efficacy in NSCLC with KRAS mutation.

These studies showed that targeting Nrf2 and its downstream molecules can interfere with cancer metabolisms, such as glutaminolysis and fatty acid synthesis. It may serve as an effective strategy in the treatment of NSCLC.

## Conclusion and Perspectives

**Figure 1:**
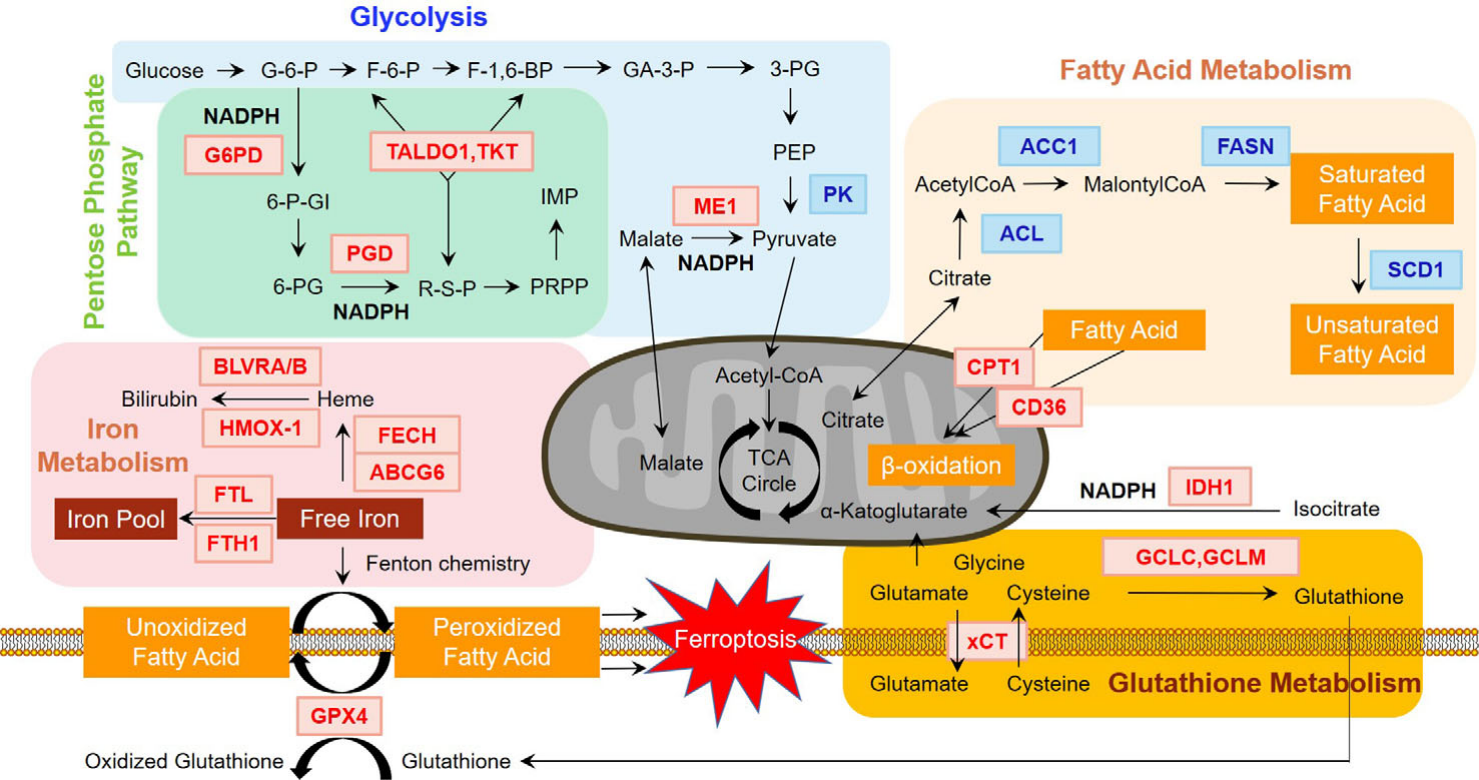
Regulation of major metabolism pathways by Nrf2. Nrf2 plays an essential role in regulating major metabolism pathways. As shown in the figure, genes in red and blue font indicate positive and negative regulation by Nrf2, respectively. In glycolysis, Nrf2 activation results in the downregulation of pyruvate kinase (PK). In pentose phosphate pathway, glucose-6-phosphate dehydrogenase (G6PD) and 6-phosphogluconate dehydrogenase (PGD) is rate-limiting enzymes, which are positively regulated by Nrf2. Nrf2 also controls the non-oxidative part of pentose phosphate pathway by increasing the levels of transaldolase 1 (TALDO1) and transketolase (TKT). In terms of NAPDH metabolism, G6PD and PGD are key enzymes of NADPH production. Nrf2 also positively regulates malic enzyme 1 (ME1) and isocitrate dehydrogenase 1 (IDH1), indicating that NADPH generation depends heavily on Nrf2. Nrf2 also participates in glutathione metabolism. Glutamate-cysteine ligase catalytic (GCLC) and modifier (GCLM) subunits regulated by Nrf2 consume NADPH in the synthesis of glutathione. Other ingredients of glutathione including glutamate and cysteine are leveled by system Xc- (xCT), another Nrf2 targeted gene, which can channel the movement of glutamate and cysteine. Iron metabolism is controlled by Nrf2 in the heme metabolism arm and iron storage arm. ATP binding cassette subfamily B member 6 (ABCG6) and Ferrochelatase (FECH) participate in heme synthesis. Biliverdin reductase A and B (BLVRA/B) and heme oxygenase (decycling) 1 (HMOX1) are enrolled in heme degradation. Ferritin light chain (FTL) and ferritin heavy chain 1 (FTH1) engage in iron pool balance. These proteins are directly or indirectly regulated by Nrf2. Ferroptosis induced by the accumulation of free iron results from peroxidized fatty acids, which can be rescued by glutathione peroxidase 4 (GPX4), regulated by Nrf2. Further, fatty acid metabolism is negatively adjusted by Nrf2. ATP-citrate lyase (ACL), acetyl-CoA carboxylase 1 (ACC1), fatty acid synthase (FASN), and stearoyl CoA desaturase 1 (SCD1) are key enzymes in fatty acid synthesis. In terms of fatty acid oxidation, Nrf2 positively regulates carnitine palmitoyltransferase 1 (CPT1) and CD36, which channel fatty acids through the membrane of mitochondria for oxidation. The influence of Nrf2 on fatty acid metabolism can alter ferroptosis, as it may affect the distribution of fatty acids.

Metabolic reprogramming of tumor cells has undergone drastic discussions during the past decade. However, the real scenario has not been fully elucidated and still needs deeper exploration. In this review, we summarize the role of Nrf2 in metabolic reprogramming and propose that Nrf2 and its downstream molecules may serve as potential therapeutic targets in NSCLC (**Figure 1**). As the main factor in response to oxidative stress, the functions of Nrf2 have been widely identified in the process of tumor growth, progression, and resistance of NSCLC. In addition, various studies have shown that Nrf2 plays an important role in the metabolic reprogramming of NSCLC, including NADPH synthesis, lipid metabolism, ROS equilibrium, glucose metabolism, mitochondrial functioning and iron activity. The crosstalk between Nrf2 and metabolic reprogramming is considered as a decisive power during NSCLC development and progression. Further studies may reveal the underlying mechanisms of Nrf2-mediated metabolic reprogramming and offer therapeutic targets for the treatment of NSCLC.

## Author Contributions

All authors listed have made a substantial, direct, and intellectual contribution to the work and approved it for publication.

## Funding

This study was funded by Major Science and Technology Special Project of Zhejiang Province (2020C03058), Zhejiang Provincial Research Center for Diagnosis and Treatment of Lung Cancer (JBZX-202007) and Key discipline of traditional Chinese medicine (integrated traditional Chinese and western medicine) in Zhejiang Province (2017YFC0113500).

## Conflict of Interest

The authors declare that the research was conducted in the absence of any commercial or financial relationships that could be construed as a potential conflict of interest.
